# Supplementary material for: Spatiotemporal organisation of protein processing in the kidney
Source: Nat Commun. 2022 Sep 29;13:5732. doi: 10.1038/s41467-022-33469-5 (PMC9522658; doi:10.1038/s41467-022-33469-5)
Supplement: Supplementary file 1 — Supplementary Information [file 41467_2022_33469_MOESM1_ESM.pdf]

Spatiotemporal organisation of protein processing in the kidney

Marcello Polesel, Monika Kaminska, Dominik Haenni, Milica Bugarski, Claus Schuh, Nevena Jankovic, Andres Kaech, Jose M Mateos, Marine Berquez & Andrew M Hall.

Supplementary Information

Supplementary figures 1-7

Supplementary table 1

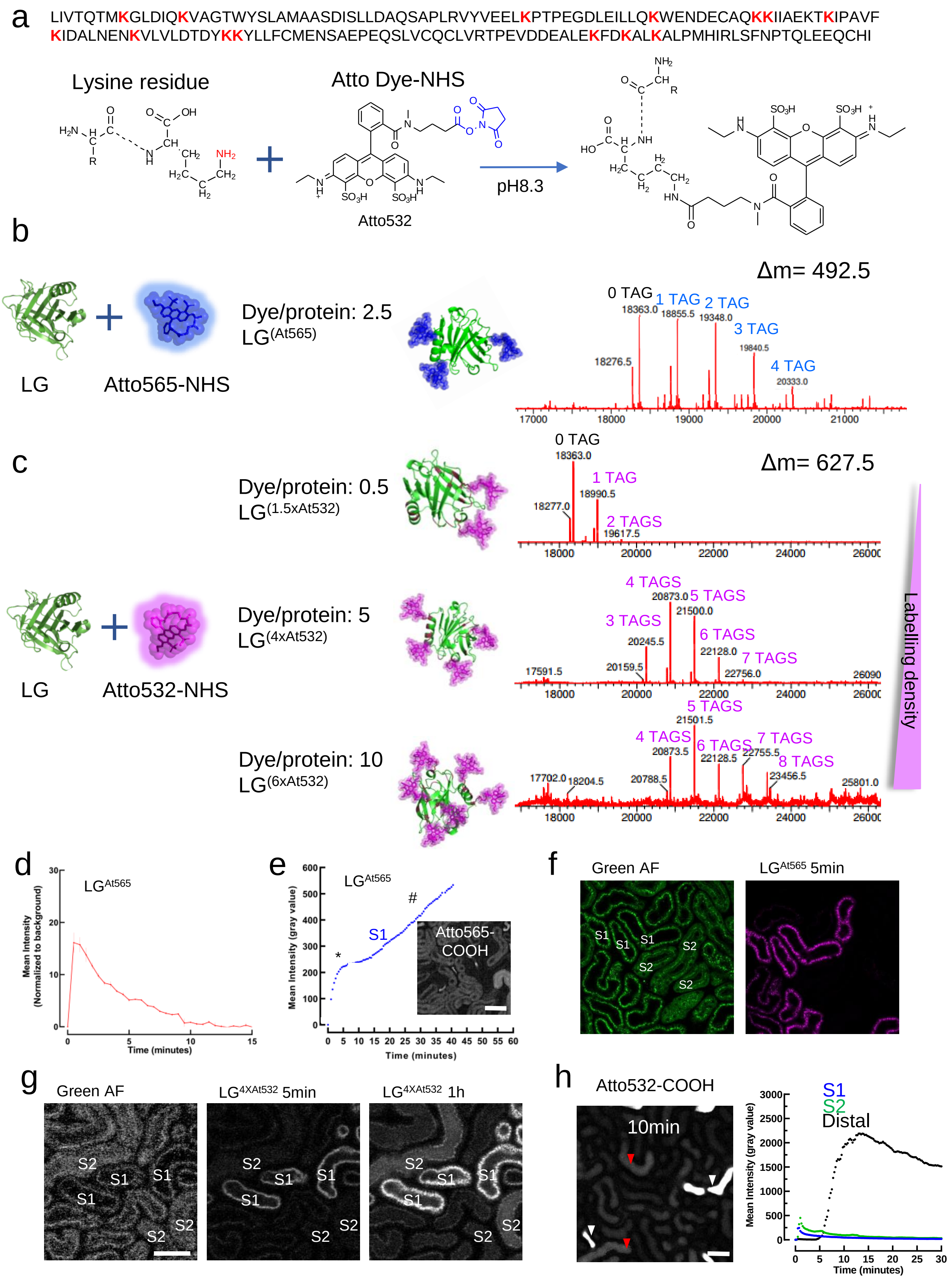

a
LIVTQTMKGLDIQKVAGTWYSLAMAASDISLLDAQSAPLRVYVEELKPTPEGDLEILLQKWENDECAQKKIIAEKTKIPAVF
KIDALNENKVLVLDTDYKKYLLFCMENSAEPEQSLVCQCLVRTPEVDDEALEKFDKALKALPMHIRLSFNPTQLEEQCHI
Lysine residue
Atto Dye-NHS
Atto532
pH8.3
b
LG
Atto565-NHS
Dye/protein: 2.5
LG(At565)
Δm= 492.5
0 TAG
1 TAG
2 TAG
3 TAG
4 TAG
c
Dye/protein: 0.5
LG(1.5xAt532)
Δm= 627.5
Dye/protein: 5
LG(4xAt532)
Dye/protein: 10
LG(6xAt532)
LG
Atto532-NHS
Labelling density
d
LGAt565
Mean Intensity (Normalized to background)
Time (minutes)
e
LGAt565
S1
Atto565-COOH
Mean Intensity (gray value)
Time (minutes)
f
Green AF
LGAt565 5min
g
Green AF
LG4XAt532 5min
LG4XAt532 1h
h
Atto532-COOH
10min
S1
S2
Distal
Mean Intensity (gray value)
Time (minutes)

Supplementary figure 1: Generation of fluorescent lactoglobulin tracers. **(a)** The sequence of lactoglobulin (LG) is reported, with lysine residues highlighted in red. Atto-NHS ester dyes were covalently bound to lysine residues by reaction at pH 8.3. (**b-c**) Labelling of LG with Atto565 or Atto532: different dye/protein ratios were used to produce low or highly labelled LG. (**d**) Fluorescence signal in the renal vasculature post intravenous injection of LG^{At565} showing rapid clearance of the protein (mean value±SEM; n=3 mice per group). (**e**) Post intravenous injection, LG^{At565} showed rapid uptake in S1 segments of the proximal tubule (*), and subsequent unquenching (#), signifying degradation. When injected alone, a small uptake of the free dye Atto565-COOH occurred in proximal tubules (picture inlay). (**f**) S1 and S2 segments were identified by green autofluorescence (AF) signals at 850nm excitation. Single plane images acquired 5 minutes post injection demonstrating uptake of LG^{At565} exclusively in S1 (representative of 3 independent experiments). (**g**) Since it was not possible to co-image green AF and $LG^{4XAt532}$, AF images were first acquired pre-injection, and the laser power was then adjusted to remove the background. A sum image of the background AF is provided, along with single plane example images showing evolution of $LG^{4XAt532}$ signals with time in S1/2 segments (representative of 3 independent experiments). (**h**) In contrast to Atto565-COOH, Atto532-COOH did not display any tubular uptake, but luminal signal was observed (red arrowheads), confirming filtration, and the dye was rapidly excreted in distal nephron segments (white arrowheads). LG labelled with Atto532 was therefore used in further experiments to assess downstream uptake of protein degradation products released from S1. Scale bars: 20µm.

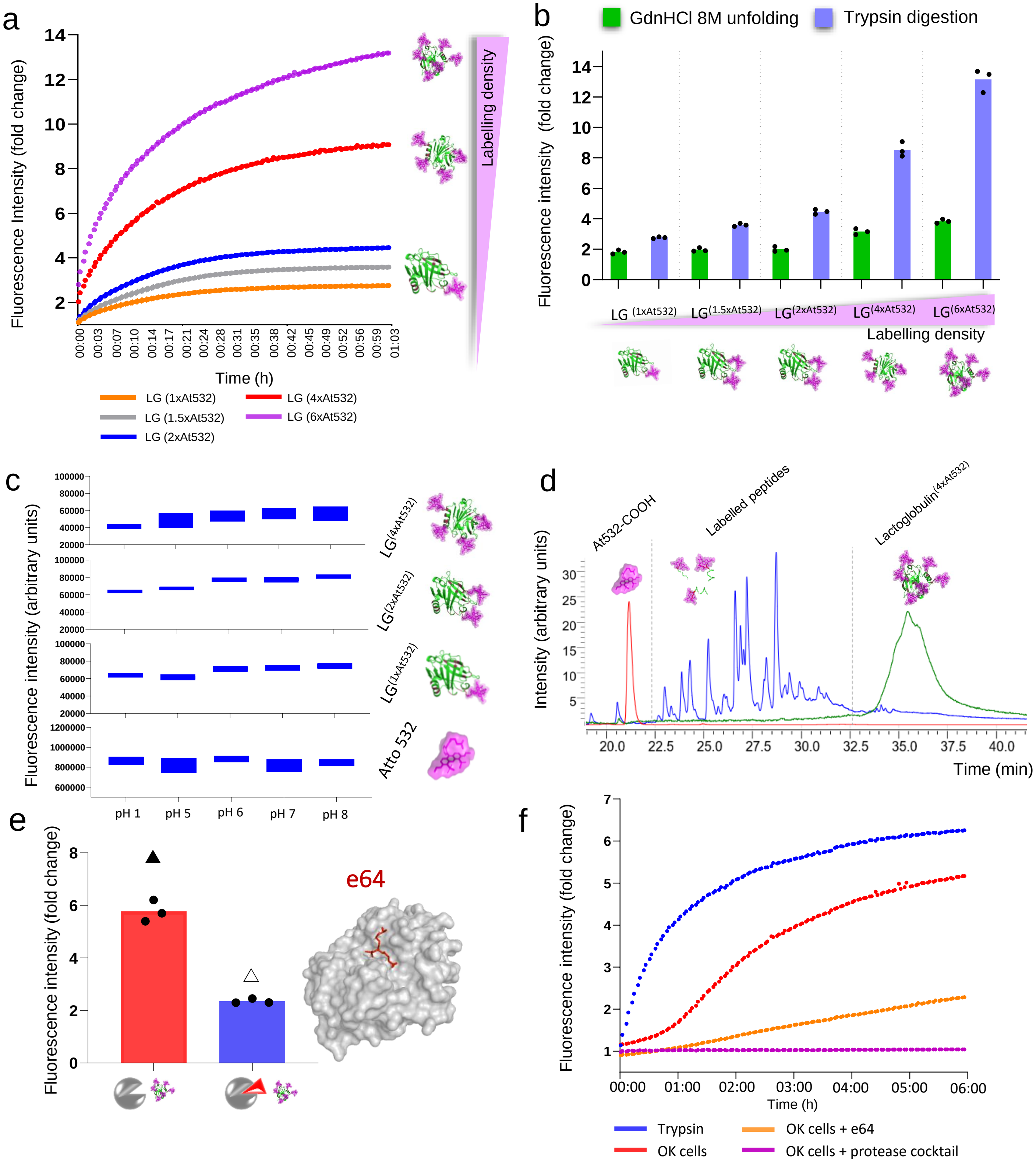

Supplementary figure 2: In vitro characterisation of labeled lactoglobulin. (**a**) Enzymatic degradation of lactoglobulin (LG) in solution with trypsin resulted in an increase in fluorescence intensity (unquenching), the magnitude of which was dependent on labelling density. Data depicted are mean values (n=3). (**b**) Comparison of the fluorescence increase induced by protein unfolding with guanidine hydrochloride (GdnHCl). Data depicted are mean values (n=3). (**c**) pH impact on LG fluorescence with different labelling densities and free dye Atto532 (data depicted are from 4 measurements per experiment, total of 2 experiments. Boxes represent the upper and lower means). (**d**) Degradation of labelled lactoglobulin by trypsin into peptides was confirmed by HPLC analysis. (**e**) Lactoglobulin$^{(4xAt532)}$ digestion by cathepsin B ▲ also caused unquenching, which was substantially reduced by the cathepsin inhibitor e64 △. Data depicted are mean values (n=3). The crystal structure of bovine cathepsin B is depicted with the inhibitor e64 (PDB-ID:1ITO). (**f**) Unquenching of lactoglobulin$^{(4xAt532)}$ by Opossum Kidney (OK) cells was inhibited by e64 and by a broad spectrum protease inhibitor cocktail (see *Methods*). Data depicted represent mean fluorescence intensity (n=2-3 experiments per condition).

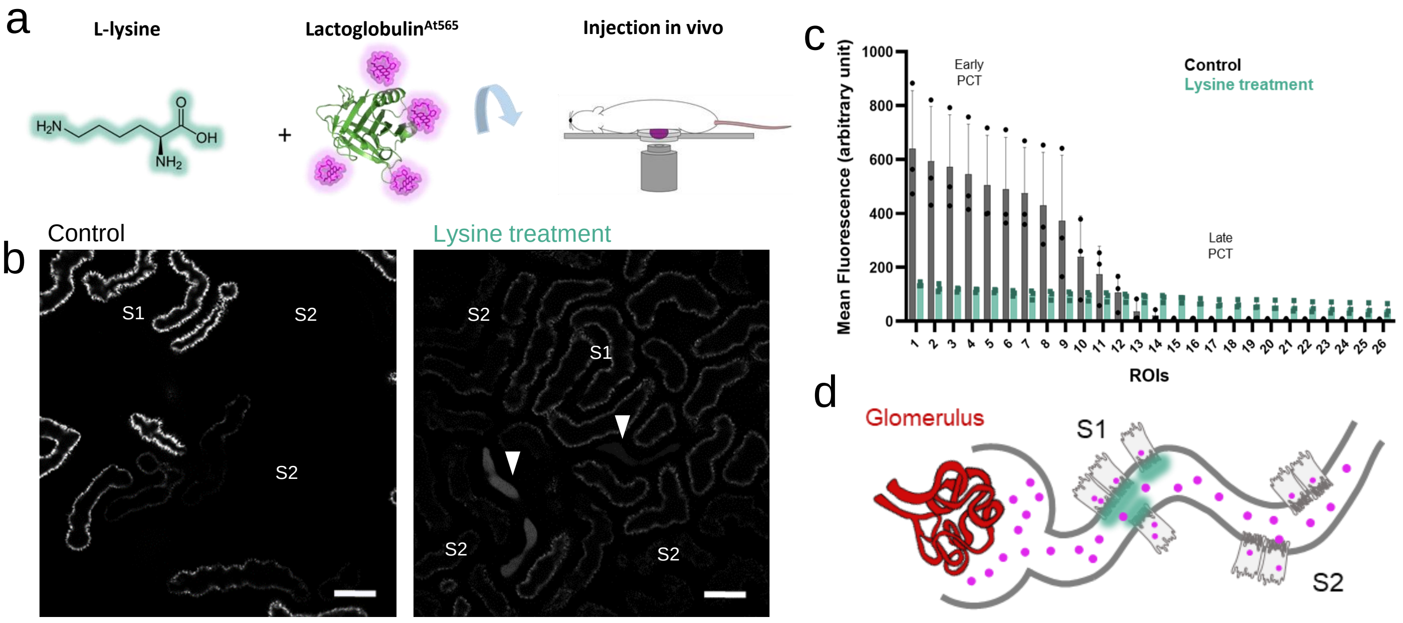

Supplementary figure 3: Uptake of filtered proteins in S2 proximal tubular segments. (**a**) To inhibit protein endocytosis, lysine was injected (2mg/g body weight) 1 minute before lactoglobulin^{At565} (25µg). (**b**) This resulted in a substantial decrease in S1 uptake, and a shift of protein reabsorption to downstream S2 segments, with some wasting in distal tubules (arrowhead) also observed. (**c**) Histogram depicting the fluorescence signal (mean value±SD; n=3 mice per group) in ROIs drawn around individual segments along the proximal convoluted tubule (PCT), ordered from highest to lowest. (**d**) Schematic representing shift of protein reabsorption to S2 when S1 uptake is inhibited. Scale bars: 20µm. Single plane example images and data depicted were acquired 10 minutes post injection of lactoglobulin.

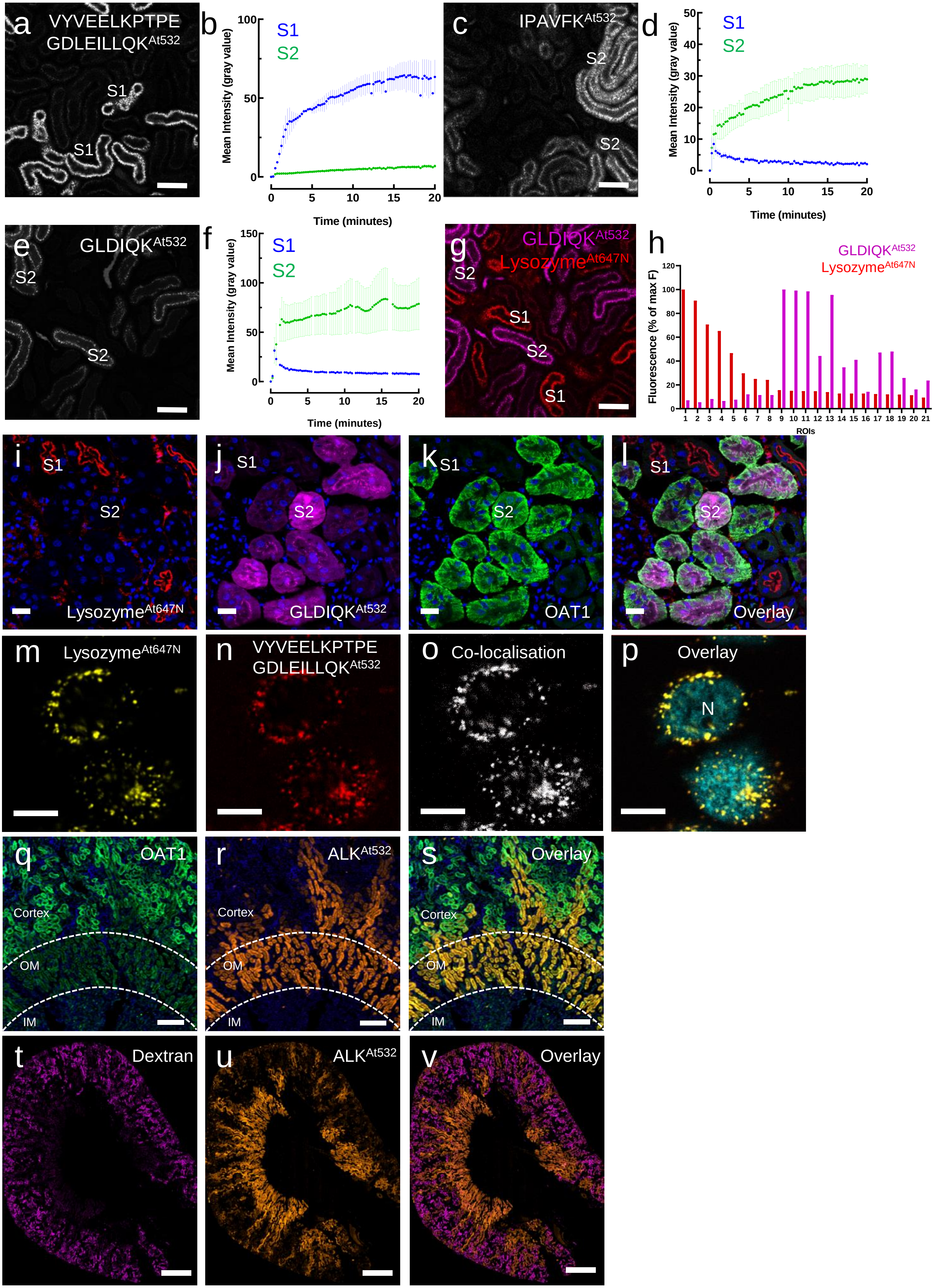

a
VYVEELKPTPE GDLEILLQK^{At532}
S1
S1
b
S1
S2
Mean Intensity (gray value)
Time (minutes)
c
IPAVFK^{At532}
S2
S2
d
S1
S2
Mean Intensity (gray value)
Time (minutes)
e
GLDIQK^{At532}
S2
S2
f
S1
S2
Mean Intensity (gray value)
Time (minutes)
g
GLDIQK^{At532}
Lysozyme^{At647N}
S2
S1
S2
S1
h
GLDIQK^{At532}
Lysozyme^{At647N}
Fluorescence (% of max F)
ROIs
i
S1
S2
Lysozyme^{At647N}
j
S1
S2
GLDIQK^{At532}
k
S1
S2
OAT1
l
S1
S2
Overlay
m
Lysozyme^{At647N}
n
VYVEELKPTPE GDLEILLQK^{At532}
o
Co-localisation
p
Overlay
N
q
OAT1
Cortex
OM
IM
r
ALK^{At532}
Cortex
OM
IM
s
Overlay
Cortex
OM
IM
t
Dextran
u
ALK^{At532}
v
Overlay

Supplementary figure 4: Tubular uptake of peptide fragments derived from in vitro degradation of lactoglobulin. (**a,b**) The large peptide VYVEELKPTPEGDLEILLQK^{At532} (2313Da) triggered uptake in S1. (**c-f**) In contrast, the small peptides IPAVFK^{At532} (674Da) and GLDIQK^{At532} (673Da) passed through S1 and were reabsorbed in S2. ROIs were drawn around tubular segments and the plots depict fluorescence intensity over time (mean value±SEM; n=3 mice per group). (**g,h**) Subsequent injection of intact lysozyme-Atto647N showed a clearly distinct uptake pattern from GLDIQK^{At532}. Single experiment showing uptake intensities along the proximal tubule, with individual ROIs ordered according to lysozyme^{Atto647N} signal intensity (from highest to lowest). Example single plane images and data depicted were acquired 20 minutes post injection. (**i-l**) Cortical region from fixed kidney tissue, showing lysozyme^{At647N} in S1 segments and GLDIQK^{At532} in segments staining positive for the S2 marker OAT1. Nuclei were labelled with Hoechst (blue). Scale bars: 20μm. Single example images are depicted representative of 3 experiments. (**m-p**) The large peptide VYVEELKPTPEGDLEILLQK^{At532} was endocytosed into PT-derived (OK) cells *in vitro*, and co-localised with intact lysozyme. Single plane example images were acquired 30 minutes after incubation (n = 3 experiments, N = nucleus labelled with Hoechst). Scale bar = 5 μm. (**q-s**) Overview of the kidney on cross-section post fixation showing expression of OAT1 in cortical S2 segments and uptake of ALK^{At532} in cortical S2 and medullary S3 segments of the proximal tubule. Scale bars: 200 μm. OM = outer medulla, IM = inner medulla. Single example images are depicted representative of 3 experiments. (**t-v**) Overview of the kidney on cross-section post fixation showing contrasting uptake patterns along the proximal tubule of a 10kDa dextran and ALK^{At532}. Scale bars: 500 μm. Single example images are depicted representative of 3 experiments.

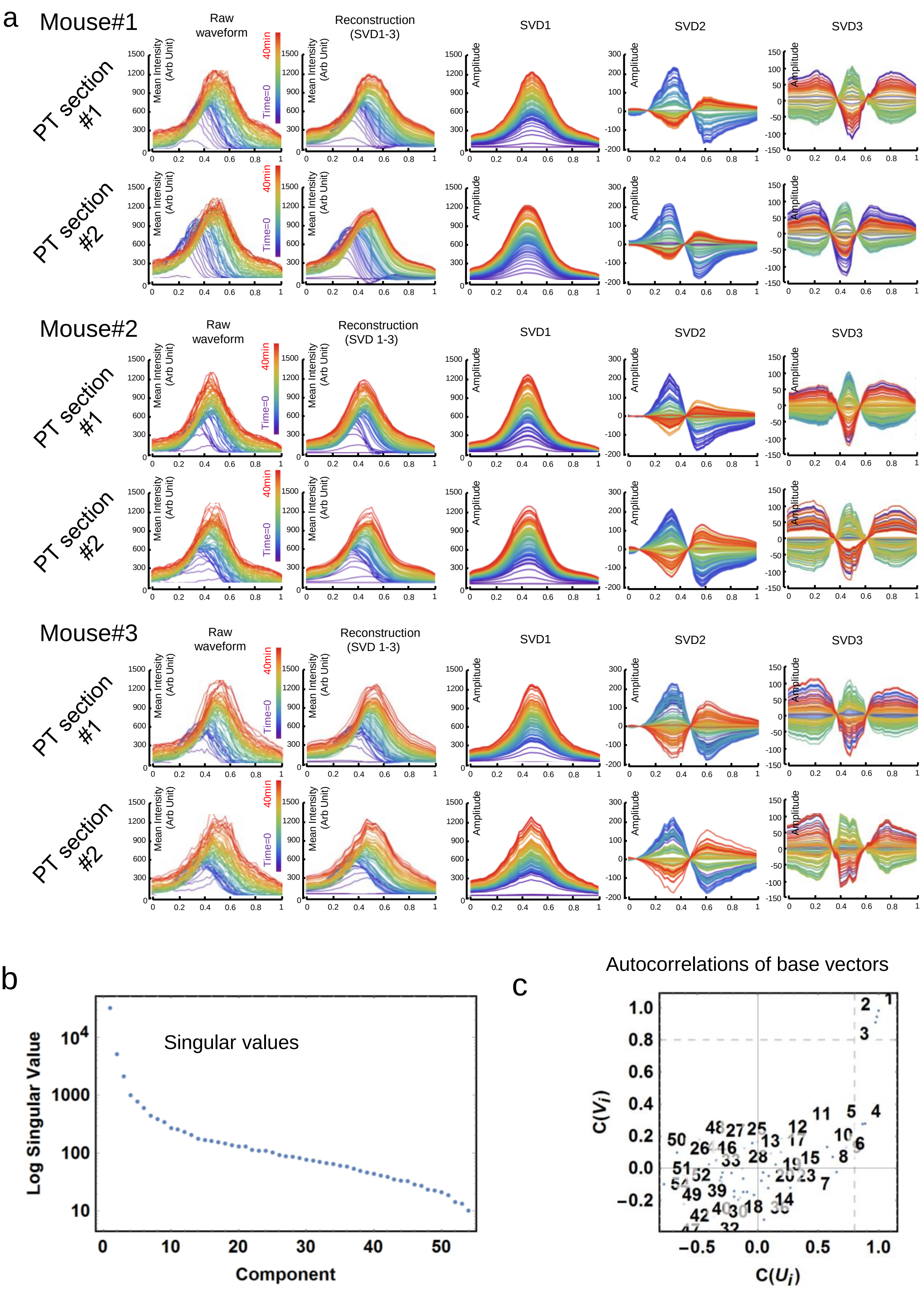
a
Mouse#1
Raw waveform
Reconstruction (SVD1-3)
SVD1
SVD2
SVD3
PT section #1
PT section #2
Mean Intensity (Arb Unit)
Amplitude
40min
Time=0
Mouse#2
Raw waveform
Reconstruction (SVD 1-3)
SVD1
SVD2
SVD3
PT section #1
PT section #2
Mouse#3
Raw waveform
Reconstruction (SVD 1-3)
SVD1
SVD2
SVD3
PT section #1
PT section #2
b
Singular values
Log Singular Value
Component
c
Autocorrelations of base vectors
C(V_i)
C(U_i)

Supplementary figure 5: Derivation of SVD base vectors and consistency of endo-lyosomal system dynamics in the proximal tubule. (**a**) Following intravenous injection with lactoglobulin^{At565}, cumulative temporal profiles of protein progression through cells in the proximal tubule (PT) were generated by line scan ROIs, which were then normalised to enable comparison (0=apical, 1=basolateral). Reconstruction of the data from the first three components of SVD analysis (see *methods*) yields a representation with reduced noise. Depicted are raw data (Raw waveform panels), reconstructed traces (Reconstruction (SVD1-3) panels), and individual base vectors are depicted from different tubular sections from 3 different mice, to demonstrate the consistency of underlying kinetics. (**b**) Singular values are depicted, showing that the first three components are clearly separated from the remaining, indicating their dominance of the signal and explaining why they can almost completely recreate the raw traces. (**c**) Autocorrelations of the columns of V (kinetic information) versus autocorrelations of the columns of U (spatial information) of the SVD of the raw data. The correlation value is indicated by the number of the component as a marker. The three major components all lie above the 0.8 threshold (dashed lines). Other components below this threshold are likely to be dominated by noise.

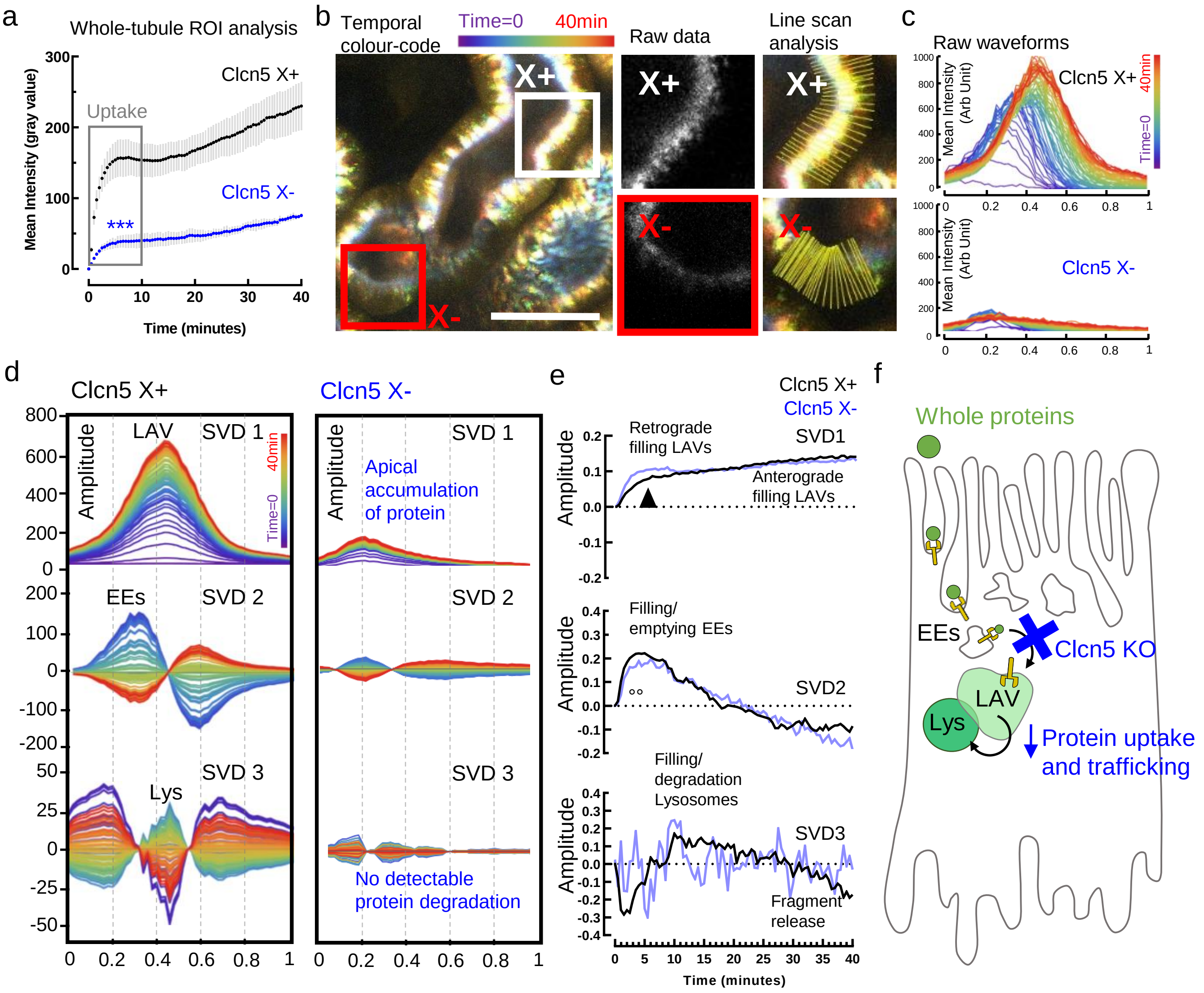

Supplementary figure 6: Functional effects of Clcn5 knockout on the endo-lysosomal system. (**a**) The plots depict fluorescence intensity over time in normal (Clcn5 X+) and affected (Clcn5 X-) proximal tubular cells, post intravenous injection of lactoglobulin^{At565} (mean value±SEM; ***p<0.001; n≥2 mice per group), in female Clcn5 knockout mice, which have a mosaic expression of the X-linked gene. (**b**) Representative temporal colour coded images depicting normal (white square) and affected (red square) regions, and the application of line scan analysis. Scale bar: 20μm. (**c**) Normalised raw trajectories of protein progression across the cell derived from line scans (0=apical, 1=basolateral). (**d**) Base vectors (1-3) derived from SVD analysis (LAV=large apical vacuole, EE=early endosome, Lys=lysosome). In affected cells, protein accumulated in the apical portion of cells, signifying a severe trafficking defect, and lysosomal protein degradation was no longer detected. (**e**) Representative kinetic plots of the base vectors. Affected cells displayed delayed transition through EEs (SVD2, time x genotype °°p<0.01), and also an apparent increase in kinetics of retrograde LAV filling (arrowhead), likely an artefact arising from the grossly altered SVD1 shape. The normal kinetic evolution of SVD3 was completely abolished in affected cells, as shown by the noisy trajectory. (**f**) Summary diagram: loss of Clcn5 results in a severe apical trafficking defect, resulting in a decreased delivery of filtered whole proteins to lysosomes for degradation.

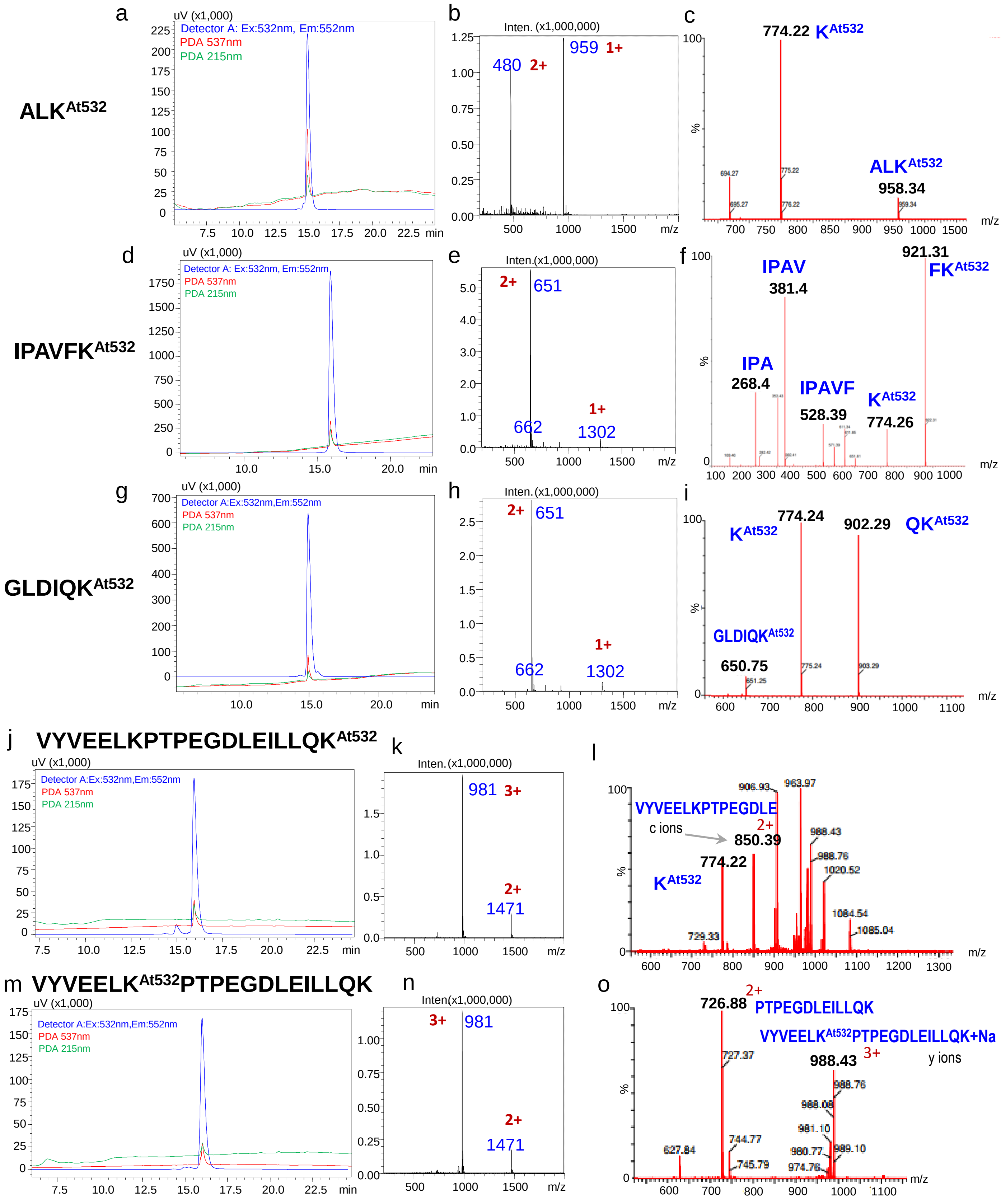

Supplementary figure 7: Characterisation of labeled peptides. (**a, d, g, j, m**) High pressure liquid chromatography (HPLC) with UV (PDA 215 nm for peptides and PDA 537 nm for Atto532) and fluorescence detection (detector A excitation: 532nm, emission: 552nm) were used to confirm the purity of peptides used in the study. (**b, e, h, k, n**) Peptide mass was confirmed by in-line mass spectrometry (MS) purity profiles. (**c, f, i, l, o**) Labeling position of Atto 532 on each peptide was confirmed with electrospray ionization MS (ESI-MS/MS) analysis.

Protein	Dye	Dye/Protein reaction ratio	Labelling density (protein mix obtained)	Tracer name
Bovine beta-lactoglobulin	Atto565	2.5	Lactoglobulin-Atto565 tags: (**+1**, **+2**, +3, +4)	Lactoglobulin^{At565}
Bovine beta-lactoglobulin	Atto532	0.3	Lactoglobulin-Atto532 tags: (0, **+1**)	Lactoglobulin$^{(1xAt532)}$
Bovine beta-lactoglobulin	Atto532	0.5	Lactoglobulin-Atto532 tags: (0, **+1, +2**)	Lactoglobulin$^{(1,5xAt532)}$
Bovine beta-lactoglobulin	Atto532	1.0	Lactoglobulin-Atto532 tags: (0, +1, **+2**)	Lactoglobulin$^{(2xAt532)}$
Bovine beta-lactoglobulin	Atto532	5	Lactoglobulin-Atto532 tags: (+3, **+4**, +5, +6)	Lactoglobulin$^{(4xAt532)}$
Bovine beta-lactoglobulin	Atto532	10	Lactoglobulin-Atto532 tags: (+4, +5, **+6**, +7, +8)	Lactoglobulin$^{(6xAt532)}$
Human lysozyme	Atto647N	10	Lysozyme-Atto647N tags: (**+1**)	Lysozyme^{At647N}
Human beta-2-microglobulin	Atto532	5	Beta-2-microglobulin-Atto532 tags: (**+1**, **+2**, +3, +4)	β2microglobulin^{At532}

Supplementary Table 1. List of fluorescently-labeled protein tracers generated after lysine conjugation of NHS-esters forms of Atto dyes. The predominant labeling densities are highlighted in bold.
