# Supplementary material for: Spatiotemporal organisation of protein processing in the kidney
Source: Nat Commun. 2022 Sep 29;13:5732. doi: 10.1038/s41467-022-33469-5 (PMC9522658; doi:10.1038/s41467-022-33469-5)
Supplement: Supplementary file 3 — Reporting Summary [file 41467_2022_33469_MOESM3_ESM.pdf]

nature portfolio

Corresponding author(s): Andrew Hall

Last updated by author(s): May 3, 2022

Reporting Summary

Nature Portfolio wishes to improve the reproducibility of the work that we publish. This form provides structure for consistency and transparency in reporting. For further information on Nature Portfolio policies, see our Editorial Policies and the Editorial Policy Checklist.

Statistics

For all statistical analyses, confirm that the following items are present in the figure legend, table legend, main text, or Methods section.

n/a	Confirmed	
☐	☒	The exact sample size (*n*) for each experimental group/condition, given as a discrete number and unit of measurement
☐	☒	A statement on whether measurements were taken from distinct samples or whether the same sample was measured repeatedly
☐	☒	The statistical test(s) used AND whether they are one- or two-sided<br>*Only common tests should be described solely by name; describe more complex techniques in the Methods section.*
☐	☒	A description of all covariates tested
☐	☒	A description of any assumptions or corrections, such as tests of normality and adjustment for multiple comparisons
☐	☒	A full description of the statistical parameters including central tendency (e.g. means) or other basic estimates (e.g. regression coefficient) AND variation (e.g. standard deviation) or associated estimates of uncertainty (e.g. confidence intervals)
☐	☒	For null hypothesis testing, the test statistic (e.g. F, t, r) with confidence intervals, effect sizes, degrees of freedom and P value noted<br>*Give P values as exact values whenever suitable.*
☒	☐	For Bayesian analysis, information on the choice of priors and Markov chain Monte Carlo settings
☒	☐	For hierarchical and complex designs, identification of the appropriate level for tests and full reporting of outcomes
☒	☐	Estimates of effect sizes (e.g. Cohen's *d*, Pearson's *r*), indicating how they were calculated

Our web collection on statistics for biologists contains articles on many of the points above.

Software and code

Policy information about availability of computer code

Data collection	Mass spectrometry was performed with Synapt G2 Si (Waters) or LCMS-2020 (Shimadzu) machines. Nano ESI-MS analysis was performed on a Synapt G2_Si mass spectrometer (Waters). Plate assays were performed on a Synergy 2 plate reader (Biotec). Intravital imaging was performed on a custom-built multiphoton microscope with an InSight DS Dual laser, Spectraphysics. Images were acquired using ScanImage software. EM images were acquired using a Zeiss Auriga 40 Crossbeam system.
Data analysis	Line scan analysis from in vivo images was performed using Wolfram Mathematica. Analysis of EM images was performed using MAPS 3 software. Segmentation of FIB-SEM images was performed using Ilastik 1.0. Mass spectrometry analysis was performed using LabSolution and MagTran software (version 1.0). Nano ESI-MS analysis was performed using MassLynx 4.2 Software (Waters).<br>Image analysis and quantification of fluorescence were performed using ImageJ. Plots and graphs, as well as statistical analyses were performed using Graphpad Prism. The code used in this study is available at a public repository (https://osf.io/qfzdt/?view_only=4a5d0b4d6bd847278a3d4cca95b1f00b).

For manuscripts utilizing custom algorithms or software that are central to the research but not yet described in published literature, software must be made available to editors and reviewers. We strongly encourage code deposition in a community repository (e.g. GitHub). See the Nature Portfolio guidelines for submitting code & software for further information.

March 2021

Data

Policy information about availability of data

All manuscripts must include a data availability statement. This statement should provide the following information, where applicable:

- Accession codes, unique identifiers, or web links for publicly available datasets
- A description of any restrictions on data availability
- For clinical datasets or third party data, please ensure that the statement adheres to our policy

Source data is provided with this paper.

Field-specific reporting

Please select the one below that is the best fit for your research. If you are not sure, read the appropriate sections before making your selection.

☒ Life sciences ☐ Behavioural & social sciences ☐ Ecological, evolutionary & environmental sciences

For a reference copy of the document with all sections, see nature.com/documents/nr-reporting-summary-flat.pdf

Life sciences study design

All studies must disclose on these points even when the disclosure is negative.

Sample size	No sample size calculation was performed - the sample size was chosen based on our previous experience of performing similar experiments. Sample sizes were sufficient to detect significant changes. A minimum of 3 mice was used in each experimental group.
Data exclusions	No data were excluded.
Replication	Experiments were performed as triplicates as a minimum. Information concerning replicates for each experiment is provided in the paper. All replication attempts were successful.
Randomization	Mice were randomly assigned to experimental groups.
Blinding	Experiments were performed in their entirety by single investigators, which precluded the possibility to be fully blinded.

Reporting for specific materials, systems and methods

We require information from authors about some types of materials, experimental systems and methods used in many studies. Here, indicate whether each material, system or method listed is relevant to your study. If you are not sure if a list item applies to your research, read the appropriate section before selecting a response.

Materials & experimental systems

n/a	Involved in the study	
☐	☒	Antibodies
☐	☒	Eukaryotic cell lines
☒	☐	Palaeontology and archaeology
☐	☒	Animals and other organisms
☒	☐	Human research participants
☒	☐	Clinical data
☒	☐	Dual use research of concern

Methods

n/a	Involved in the study	
☒	☐	ChIP-seq
☒	☐	Flow cytometry
☒	☐	MRI-based neuroimaging

Antibodies

Antibodies used	The following primary antibodies were used for immunofluorescence:<br>rabbit anti-Rab11 (D4F5) (1:100, Cell Signalling Technology, #5589), rat anti-Lamp1 (1D4B) (1:100, Abcam, ab25245), mouse anti-Lrp2 (CD7D5) (1:200, Novus Biologicals, NB110-96417), rabbit anti-OAT1 (Alpha Diagnostic International, OAT11-A), goat anti-Cathepsin L (1:100, R&D Systems, AF1515-SP), rabbit anti-OAT1 (1:500, Alpha Diagnostic International, OAT11-A).<br>The following secondary conjugated antibodies were used (all at 1:500): Dylight488 goat anti-rat (Bethyl Laboratories/Lubioscience, A110-242D2, a kind gift from Prof Sommer, University of Zurich), Alexa647 donkey anti-rabbit (Jackson ImmunoResearch, 711-606-152), Alexa488 donkey anti-rabbit (Jackson ImmunoResearch, 711-546-152), Alexa647 donkey anti-goat (Jackson ImmunoResearch, 705-606-147), AbberiorStar635-P goat anti-mouse (kind gift from The Center for Microscopy and Image analysis, University of Zurich, comercially available from Thermofisher).<br>Brush-border actin filaments were stained with ActinRed 555 ReadyProbes reagent (Invitrogen, R37112).

Validation	All of the listed antibodies are commercially available and have been verified by the manufacturer. All primary antibodies were validated by the relevant manufacturer for immuno-staining and usage in mouse tissue, with the exception of the OAT1 Ab, which was used in a previous study (PMID: 32062662), and displayed a staining pattern typical for OAT1 in the kidney.

Eukaryotic cell lines

Policy information about cell lines

Cell line source(s)	Opossum Kidney (OK) cells were a kind gift from Prof Olivier Devuyst, University of Zurich (commercially available from atcc.org: CRL-1840).
Authentication	The epithelial nature of OK cells was confirmed by visual inspection of cell morphology. Endocytosis activity was confirmed by uptake of fluorescently labeled proteins.
Mycoplasma contamination	Testing for mycoplasma contamination was negative.
Commonly misidentified lines (See ICLAC register)	No commonly misidentified cells were used in the study.

Animals and other organisms

Policy information about studies involving animals; ARRIVE guidelines recommended for reporting animal research

Laboratory animals	Experiments were performed on male C57Bl/6JRj mice (supplied by Janvier, Le Genest, France). The OcrlY/- mice used in this study were generated previously by the group of Prof Olivier Devuyst, University of Zurich, and have a mixed background (C57Bl/6, 129/Sv). Knockout of Ocrl alone in mice does not produce a kidney phenotype, due to compensation from INPP5B, a close paralogue. Mice were therefore generated lacking Ocrl, but expressing human INPP5B, to provide a humanized background. Male OcrlY/-mice were used in the present study. Experiments using Clcn5 deficient mice were performed in females, which have a mosaic expression of the X-linked gene and a C57Bl/6 background. For all experiments, mice were used between the ages of 6-12 weeks. All mice were housed in the University of Zurich animal facility, at ambient temperature and on a 12 hour light cycle, with free access to food (Kliba Nafag formula 3436) and water.
Wild animals	No wild animals were used in the study.
Field-collected samples	No field collected samples were used in the study.
Ethics oversight	The research in this study complies with all relevant ethical regulations, and the protocols for animal experiments were approved by The Zurich Cantonal Veterinary Office (ZH194/16).

Note that full information on the approval of the study protocol must also be provided in the manuscript.
